# Extending the phenotypic spectrum of *PRPF8*, *PRPH2, RP1* and *RPGR*, and the genotypic spectrum of early-onset severe retinal dystrophy

**DOI:** 10.1186/s13023-021-01759-8

**Published:** 2021-03-12

**Authors:** Michalis Georgiou, Naser Ali, Elizabeth Yang, Parampal S. Grewal, Tryfon Rotsos, Nikolas Pontikos, Anthony G. Robson, Michel Michaelides

**Affiliations:** 1 UCL Institute of Ophthalmology, University College London, 11-43 Bath Street, London, EC1V 9EL UK; 2 Moorfields Eye Hospital, London, UK; 3 First Division of Ophthalmology, General Hospital of Athens, National and Kapodistrian University of Athens, Athens, Greece

**Keywords:** *PRPF8*, *PRPH2*, *RP1*, *RPGR*, Early onset retinal dystrophy, LCA, Leber congenital amaurosis, Childhood blindness, Inherited retinal dystrophy, Severe early childhood onset retinal dystrophy, SECORD, EOSRD

## Abstract

**Purpose:**

To present the detailed retinal phenotype of patients with Leber Congenital Amaurosis/Early-Onset Severe Retinal Dystrophy (LCA/EOSRD) caused by sequence variants in four genes, either not (n = 1) or very rarely (n = 3) previously associated with the disease.

**Methods:**

Retrospective case series of LCA/EOSRD from four pedigrees. Chart review of clinical notes, multimodal retinal imaging, electrophysiology, and molecular genetic testing at a single tertiary referral center (Moorfields Eye Hospital, London, UK).

**Results:**

The mean age of presentation was 3 months of age, with disease onset in the first year of life in all cases. Molecular genetic testing revealed the following disease-causing variants: *PRPF8* (heterozygous c.5804G > A), *PRPH2* (homozygous c.620_627delinsTA, novel variant), *RP1* (homozygous c.4147_4151delGGATT, novel variant) and *RPGR* (heterozygous c.1894_1897delGACA). *PRPF8, PRPH2,* and *RP1* variants have very rarely been reported, either as unique cases or case reports, with limited clinical data presented. *RPGR* variants have not previously been associated with LCA/EOSRD. Clinical history and detailed retinal imaging are presented.

**Conclusions:**

The reported cases extend the phenotypic spectrum of *PRPF8-*, *PRPH2-, RP1-*, and *RPGR-*associated disease, and the genotypic spectrum of LCA/EOSRD. The study highlights the importance of retinal and functional phenotyping, and the importance of specific genetic diagnosis to potential future therapy.

**Supplementary Information:**

The online version contains supplementary material available at 10.1186/s13023-021-01759-8.

## Introduction

Leber Congenital Amaurosis/Early-Onset Severe Retinal Dystrophy (LCA/EOSRD) are genetically and phenotypically heterogeneous groups of inherited retinal diseases, with widely overlapping features. Clinical presentation includes severe congenital/early infancy visual loss, nystagmus, amaurotic pupils and a markedly abnormal or undetectable full-field electroretinogram (ERG) [1]. The most common causative genes are *CEP290* [2], *GUCY2D* [3], *CRB1* [4], and *RPE65* [5–8]*.* In total, the reported LCA/EOSRD-associated genes (n = 25) account for approximately 70–80% of cases [6, 7], with more genes yet to be identified.

Advances in ocular genetics, retinal imaging and molecular biology, have been pivotal in developing treatments for inherited retinal diseases. The first FDA- and EMA-approved gene therapy is available for LCA/EOSRD-associated with *RPE65* [9, 10]*,* and there are multiple other trials underway for LCA/EOSRD and other inherited retinal diseases. LCA/EOSRD is a disease with arguably the poorest visual prognosis. It is of paramount importance to molecularly characterize LCA/EOSRD patients, in order to facilitate access to, and potential benefit from, the on-going advances in the field.

Herein we describe the detailed retinal phenotype of five cases, from four unrelated families, with LCA/EOSRD harboring disease-causing sequence variants in genes not usually associated with the disease. We thereby extend the phenotypic spectrum associated with *PRPF8*, *PRPH2, RP1*, and *RPGR*, and the genotypic spectrum of LCA/EOSRD.

## Methods

### Patient identification

Patients were identified from the Moorfields Eye Hospital Inherited Eye Disease database. Patients were included in this database after obtaining informed consent. This retrospective study adhered to the tenets of the Declaration of Helsinki and was approved by the Moorfields Eye Hospital ethics committee.

### Clinical assessment

Medical notes and clinical images were reviewed. This included results of comprehensive ophthalmic clinical assessment, including dilated fundoscopy. Multimodal imaging was reviewed, including color fundus photography, optical coherence tomography (OCT) and fundus autofluorescence (FAF) imaging. The age of disease onset was defined as the age of the first disease related symptom(s).

### Electrophysiology assessment

Full-field and pattern electroretinography (ERG) were performed to incorporate the International Society for Clinical Electrophysiology of Vision (ISCEV) standards using gold foil corneal (n = 2) or lower eyelid skin recording electrodes (n = 1) [11, 12]. The flash ERGs in the youngest children (n = 2) were performed using non-Ganzfeld flash stimuli and lower eyelid skin electrodes according to a modified protocol [13].

### Genetic testing

Molecular genetic testing with panel screening for 176 genes known to be implicated in retinal dystrophies (Manchester Centre for Genomic Medicine) was performed in all patients, followed by Sanger sequencing of the candidate gene. Minor allele frequency for the identified variants in the general population was assessed in the Genome Aggregation Database (gnomAD) datasets (http://gnomad.broadinstitute.org/). The Combined Annotation Dependent Depletion (CADD) score was calculated for all variants; a score greater than 15 is usually considered as mildly pathogenic and a score above 20 is strongly indicative [14]. Segregation was performed in all cases (Tables 1 and Additional file 1: Table).

**Table 1:** Demographics and genetics

ID	Sex	Gene	Pedigree	c.DNA	Protein	Zygosity	Inheritance mode	Phenotype	Age of onset	Presenting symptom
P1	F	*PRPF8*	GC23684	c.5804G > A	p.Arg1935His	Heterozygous	Autosomal Dominant	EOSRD	At Birth	Decreased vision
P2	F	*PRPH2*	GC21703	**c.620_627delinsTA**	**p.Asp207_Gly208del**	Homozygous	Autosomal Recessive	EOSRD	At Birth	Nystagmus
P3^‡^	F	*RP1*	GC21938	**c.4147_4151delGGATT**	p.Gly1383*	Homozygous	Autosomal Recessive	EOSRD	6 months	Nystagmus
P4^‡^	M	*RP1*	GC21938	**c.4147_4151delGGATT**	p.Gly1383*	Homozygous	Autosomal Recessive	EOSRD	7 months	Nyctalopia
P5^†^	F	*RPGR*	GC17432	c.1894_1897delGACA	p.Asp632Lysfs*4	Heterozygous	X-linked	LCA/EOSRD	4 months	Nystagmus
C1^†^	F	*RPGR*	GC17432	c.1894_1897delGACA	p.Asp632Lysfs*4	Heterozygous	X-linked	Carrier	NA	Asymptomatic Screening at X years old

*F* female, *M* male, *NA* not available, *LCA* leber congenital amaurosis, *EOSRD* early-onset severe retinal dystrophy

The variants in **bold** are novel. ^‡^Siblings, ^†^P5 is mother of C1

## Results

Five patients (4 females) from four families were included in our study. The mean age at presentation was 3 months of age (with 2 patients being symptomatic at birth). Each individual patient and genotype are presented in detail below. Table 1 summarizes their demographics and genetics. All variants are predicted to be pathogenic [14]. Additional file 1: table presents the annotation of the variants and segregation.

### *PRPF8*-EOSRD: Patient 1

Patient 1 had poor vision since birth and especially poor vision in dim illumination from the age of 12 months. Her peripheral and central vision slowly deteriorated over the first decade of life. She was otherwise healthy, with no known family history of consanguinity, nor any history of inherited eye disease.

#### Clinical findings

**Fig. 1:**
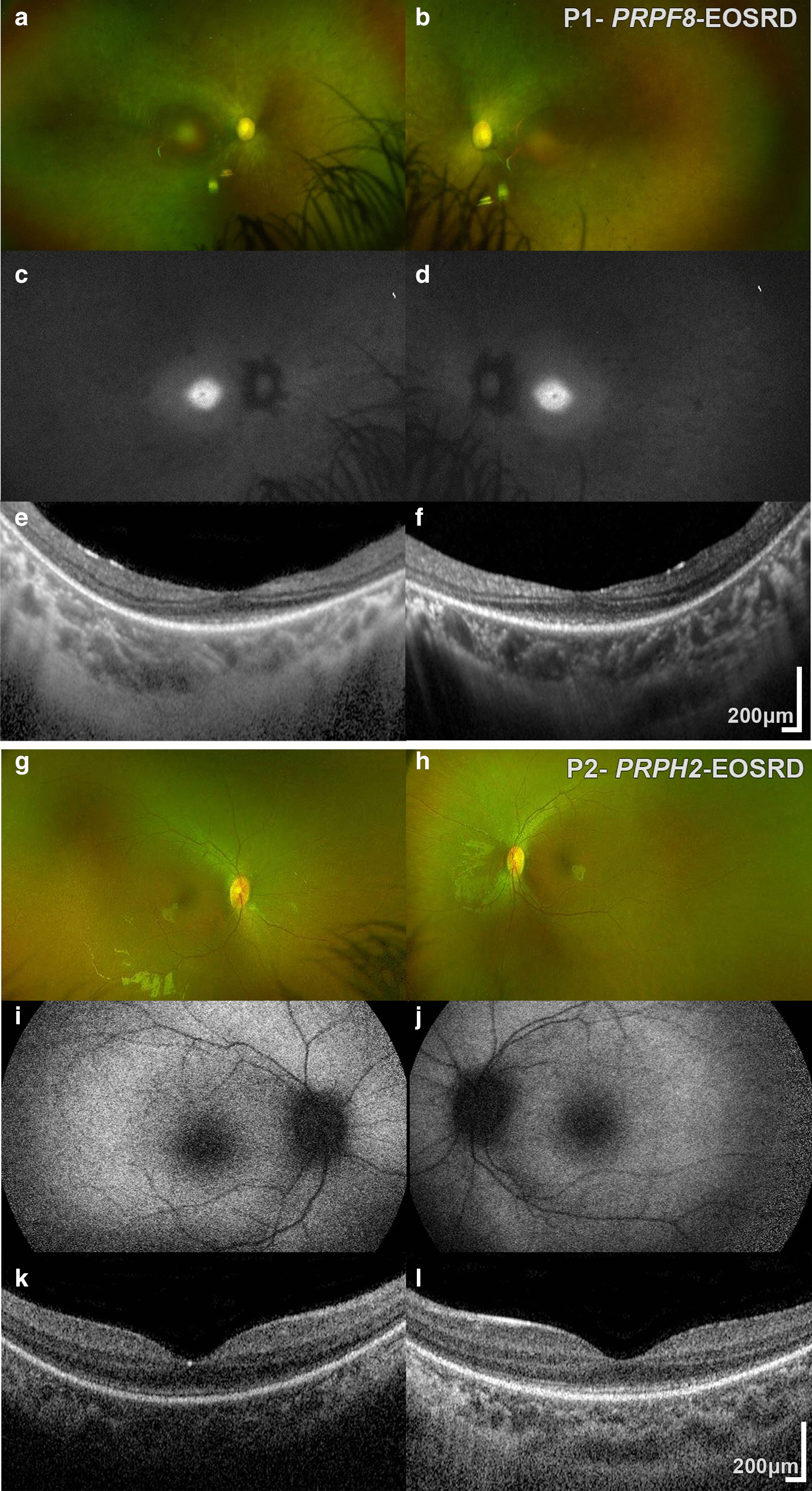
Multimodal imaging of *PRPF8* and *PRPH2* Early-Onset Severe Retinal Dystrophy (EOSRD). Multimodal imaging of: **a**–**f** Patient 1 with *PRPF8*-EOSRD and (**g**–**l**) Patient 2 with *PRPH2*-EOSRD. Patient 1: **a**, **b** Color Fundus Photographs (CFP) showing bilateral optic disc pallor, generalised retinal vascular attenuation, with retinal bone spicule formation in the periphery, **c**, **d** fundus autofluorescence (FAF) imaging with diffuse decreased signal and increased signal at the fovea, and **e**–**f** Optical Coherence Tomography (OCT) imaging showing diffuse loss of the ellipsoid zone (EZ), with residual EZ observed only at the foveal center, and preservation of the inner retinal layers. Patient 2: **g**–**h** CFP showing minimal mid-peripheral pigment migration, **i**–**j** FAF imaging with a relatively normal pattern, and **k**–**l** OCT imaging with loss of the EZ beyond the foveal center and preservation of the inner retinal layers

At 14 years old, best corrected visual acuity (BCVA) was 1.0 LogMAR on the right and 0.80 LogMAR on the left. Anterior segment examination showed bilateral posterior subcapsular lens opacities. Dilated fundus examination revealed bilateral optic disc pallor, generalised retinal vascular attenuation, with retinal bone spicule formation and white dots (Fig. 1a,b). Her visual fields to confrontation were approximately 10 degrees in both eyes. She later underwent cataract surgery to both eyes at 16 years old, which reduced her symptoms of glare, and only improved her BCVA to 0.60 LogMAR in the left eye. Electrophysiological assessment was not performed.

#### Retinal imaging

FAF imaging was abnormal with diffuse decreased signal and increased signal at the fovea (Fig. 1c, d). OCT imaging revealed diffuse loss of the ellipsoid zone (EZ), with attenuated residual subfoveal EZ (Fig. 1e, f). The inner retinal layers were relatively well preserved.

#### Molecular Genetics

Testing identified a heterozygous *PRPF8* variant; c.5804G > A, p.Arg1935His. *PRPF8* screening was offered to her family members, and both parents tested negative. This therefore likely represents a de novo variant or that one of the parents is mosaic for this variant at an undetectable level.

### PRPH2-EOSRD: Patient 2

Patient 2 presented at 14 months old, with poor vision, nystagmus and nyctalopia since birth, with a prominent light staring response. She was otherwise in good health.

#### Clinical findings

On presentation, she had rotatory nystagmus, with BCVA of 1.3 LogMAR using Cardiff cards at 50 cm with both eyes open. She had mild hypermetropic astigmatism. Anterior segment examination was normal. Widefield color fundus photographs showed minimal mid-peripheral pigment migration (Fig. 1g, h). By five years old, BCVA was 0.88 and 0.94 LogMAR in the right and left eye respectively, with stable ocular findings.

#### Retinal imaging

FAF imaging was relatively normal (Fig. 1i, j). OCT imaging revealed loss of the EZ beyond the foveal center and preservation of inner retinal layers (Fig. 1k, l). OCT and FAF imaging at age 28 and 25 years, for the father and the mother respectively, identified only a small drusenoid deposit in the left eye of the father, and was otherwise unremarkable.

#### Electrophysiological assessment

Non-Ganzfeld flash ERGs were performed with lower eyelid recording electrodes at 18 months of age. Scotopic bright flash ERG a-waves were undetectable and photopic 30 Hz flicker and single flash ERGs undetectable bilaterally, consistent with severe generalised rod and cone photoreceptor dysfunction.

#### Molecular genetics

Testing identified a *PRPH2* variant; c.620_627delinsTA, p.Asp207_Gly208del. Sanger sequencing data indicated she was homozygous for the variant. Her parents were first cousins of Pakistani origin. The variant was segregated to both parents, who were asymptomatic carriers.

### *RP1*-EOSRD: Patient 3 and 4

Patient 3 presented at 5 years old, with poor vision and nystagmus from 6 months old and nyctalopia from early childhood. Patient 4 is the affected sibling of Patient 3, and presented at 11 years of age, with nyctalopia since the age of 7 months. Both were otherwise healthy. There was a family history of consanguinity.

#### Clinical findings

**Fig. 2:**
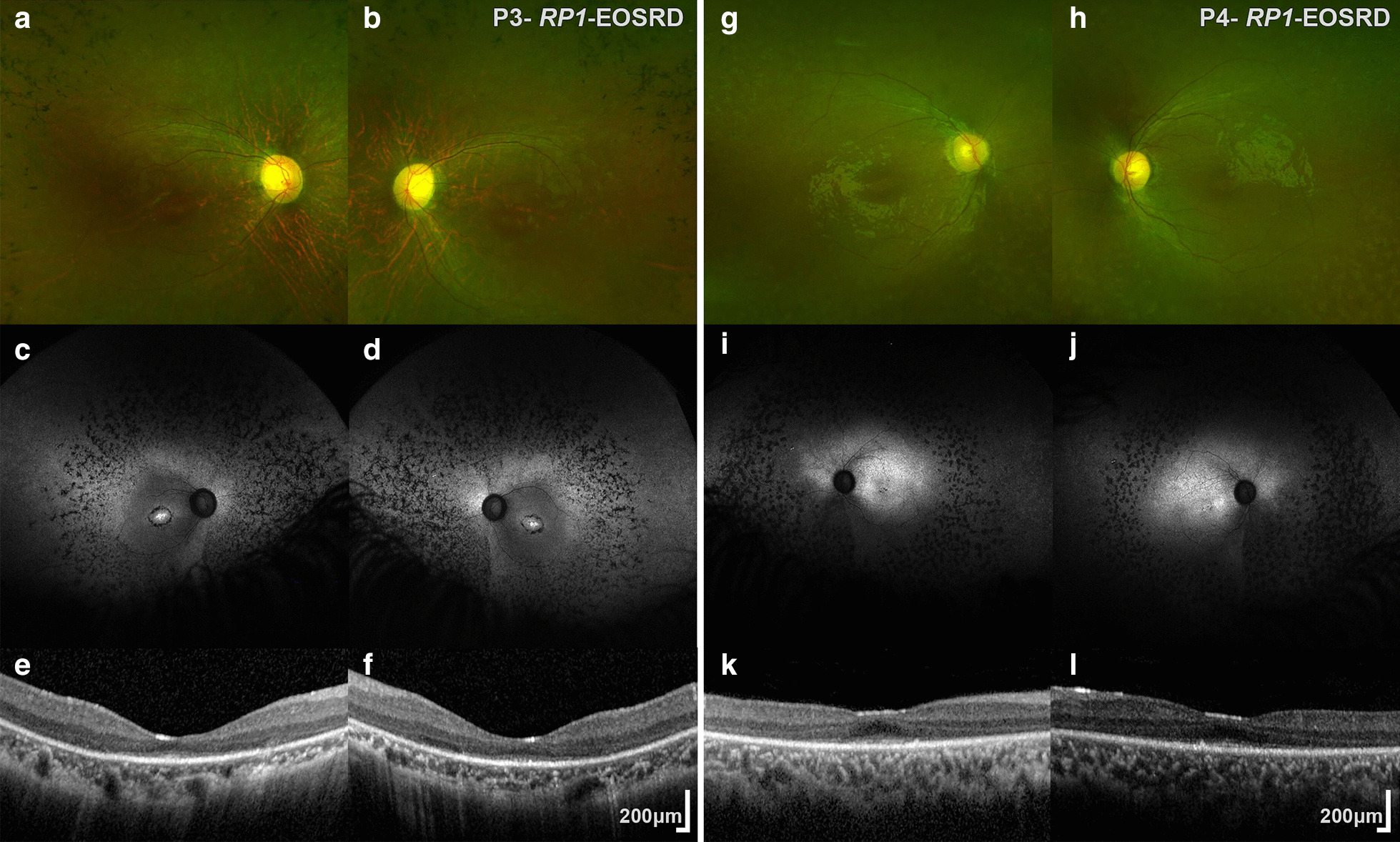
Multimodal imaging in *RP1* Early-Onset Severe Retinal Dystrophy (EOSRD). Multimodal imaging of two siblings with *RP1*-EOSRD. Patient 3: **a**, **b** Color Fundus Photographs (CFP) with bilateral macular retinal pigment epithelial (RPE) disturbance with mid-peripheral RPE migration. **g**–**h** Patient 4: CFP with bilateral macular retinal pigment epithelial disturbance (mid-peripheral RPE migration was not captured in this field of view). Optical Coherence Tomography (OCT) and fundus autofluorescence (FAF) findings were similar for both patients. **c**, **d** and **i**, **j** FAF imaging revealed bilateral peripheral signal reduction, with distinct-punctate atrophic areas in the mid-periphery and increased foveal signal. Patient 3 also had a perifoveal ring of atrophy. **e**, **f** and **k**, **l** OCT imaging demonstrated loss of outer retinal architecture and foveal hypoplasia

Patient 3 had a BCVA of 0.6 and 0.42 LogMAR in the right and left eyes respectively (at 5 years old). Cycloplegic refraction revealed a myopic and astigmatic refractive error, with constricted visual fields to approximately 10 degrees in both eyes. Dilated fundus examination revealed bilateral macular retinal pigment epithelial disturbance with mid-peripheral retinal pigment epithelial migration (Fig. 2a, b).

Patient 4 had a BCVA of 0.44 LogMAR in the right and 0.30 LogMAR in the left, with constricted visual fields to 20 degrees in both eyes (at 11 years old). Dilated fundus examination revealed bilateral macular retinal pigment epithelial disturbance with mid-peripheral retinal pigment epithelial migration (Fig. 2g, h), similar to his brother. Both patients had normal anterior segment examination with clear crystalline lenses.

#### Retinal imaging

OCT and FAF findings were similar for both patients. FAF imaging revealed bilateral peripheral signal reduction, with distinct hypoautofluorescent areas (Fig. 2c, d and i, j). OCT demonstrated loss of outer retinal architecture at the central macula in both eyes (Fig. 2e, f and k, l). Foveal hypoplasia was observed.

#### Electrophysiological assessment

In Patient 3, non-Ganzfeld flash ERGs were performed using lower eyelid skin electrodes at 5 years of age. Scotopic bright flash ERG a-waves were undetectable, and photopic 30 Hz flicker and single flash ERGs identified residual responses, with severe delay bilaterally, consistent with severe generalised rod and cone photoreceptor dysfunction. Pattern ERG recordings were also performed with skin electrodes and were almost undetectable to both a standard and large stimulus field size, in keeping with macular involvement bilaterally.

Patient 4 underwent ISCEV-standard ERG and pattern ERG testing at the age of 11 years, using corneal electrodes. The dark-adapted (DA) and light adapted (LA) responses (DA0.01, DA10.0, LA30 Hz and LA3.0 ERGs) were undetectable bilaterally, indicating a severe loss of rod and cone photoreceptor function. Pattern ERGs, were undetectable to both a standard (15 × 12 degrees) and large (30 × 24 degrees) stimulus field bilaterally, consistent with severe macular involvement.

#### Molecular genetics

The patients were homozygous for the *RP1* variant c.4147_4151delGGATT, p.Gly1383*. The variant was segregated to their mother.

### *RPGR*-LCA/EOSRD: Patient 5

Patient 5 presented with congenital nystagmus (rotatory) and markedly reduced visual acuity at 4 months old. Over time, she reported increasing nyctalopia, with gradually increasing peripheral field loss during childhood. There was a family history of poor night vision and myopia, affecting the mother and maternal grandmother. There was no family history of consanguinity. She had right squint surgery at the age of 17 years and subsequent right lateral rectus botulinum toxin injection. There was also a history of previous sinus surgery.

#### Clinical findings

**Fig. 3:**
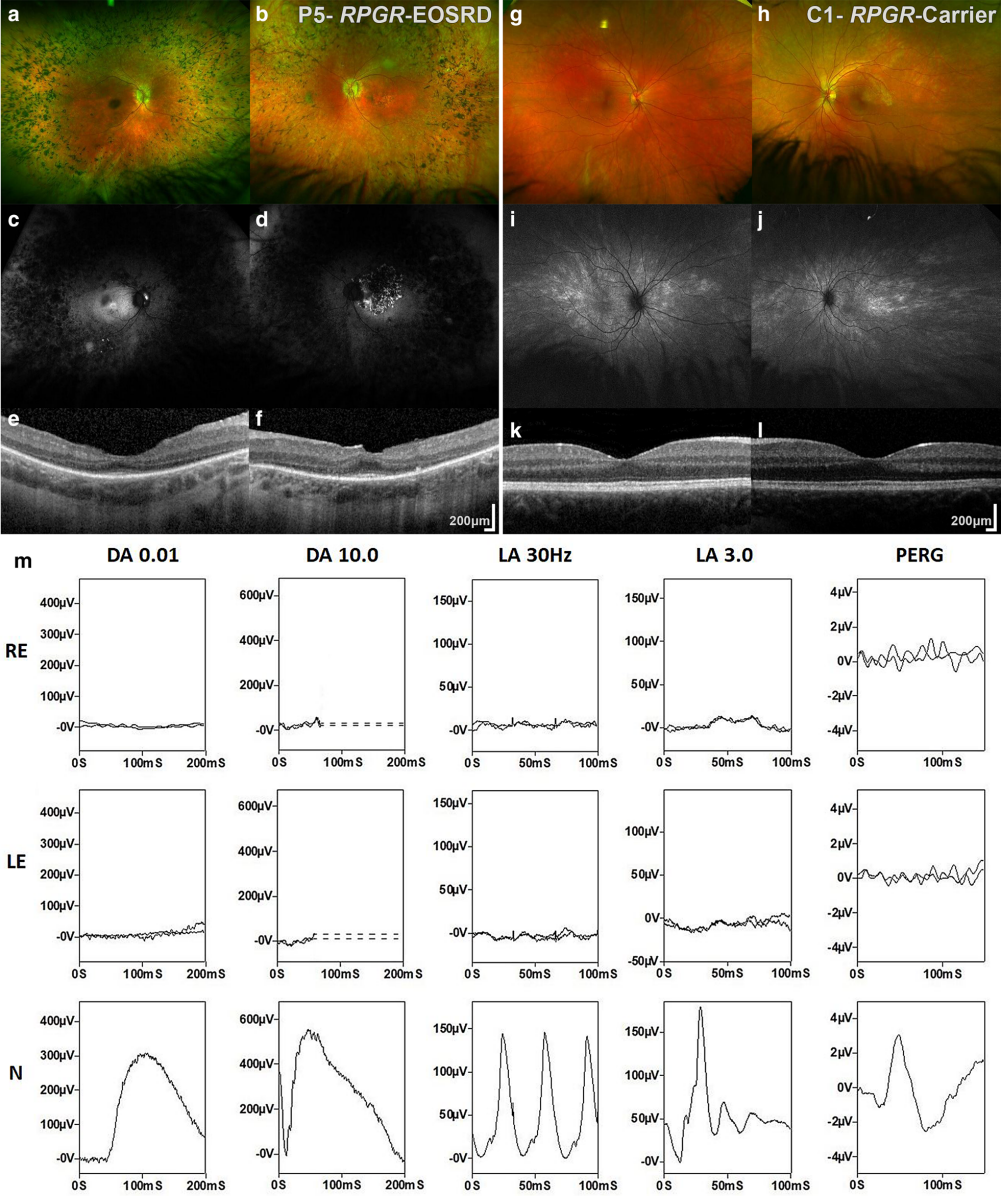
Multimodal imaging of *RPGR* Early-Onset Severe Retinal Dystrophy (EOSRD) and her RPGR Carrier daughter. Patient 5: **a**, **b** Color Fundus Photographs (CFP) with mid-peripheral bone spicule pigmentation, arteriolar attenuation and waxy disc pallor in both eyes. **c**, **d** Fundus autofluorescence (FAF) imaging with bilateral peripheral signal reduction and diffuse atrophy. **e**, **f** Loss of outer retinal architecture outside of the foveal center and foveal hypoplasia. The patient had a left choroidal neovascular membrane (**b**), which is visible on FAF as an area with a speckled appearance, of increased and decreased signal (**d**). A small part of the membrane is visible on the transfoveal optical coherence tomography (OCT), nasal to the foveal center (**f**). *RPGR*-Carrier (daughter of Patient 5): **g**, **h** CFP with slight granular RPE changes in both maculae and tapetal-like reflex (TLR) typical of X-linked carrier status, **i**–**j** FAF imaging with TLR extending radially from the fovea. **k**–**l** OCT imaging with intact structure and increased reflectivity of the outer retinal bands. **m** Full-field ERG (ffERG) and pattern ERG (PERG) recordings from the right (RE) and left (LE) eye of Patient 5, compared with recordings from a representative unaffected control subject (**n**). ffERGs are consistent with a severe photoreceptor dystrophy affecting both rods and cones. The standard PERG P50 is undetectable in keeping with severe macular involvement. Patient traces are superimposed to demonstrate reproducibility. Broken lines replace blink artefacts that occur after the DA10.0 ERG

BCVA was 1.0 LogMAR on the right and 0.82 LogMAR on the left at 22 years old. She also had rotatory nystagmus. Examination revealed mild bilateral posterior subcapsular lens opacities, and typical fundus features of retinitis pigmentosa, including mid-peripheral bone spicule pigmentation, arteriolar attenuation and waxy disc pallor bilaterally (Fig. 3a, b).

She later developed a sudden decrease in BCVA from 0.82 to 1.78 in her left eye aged 29, and was found to have developed a choroidal neovascular membrane (CNV) (Fig. 3d, f). She was treated with serial intravitreal bevacizumab injections; requiring a total of 9 injections to establish stabilization, with a BCVA of 1.0.

#### Retinal imaging

At 29 years old, FAF imaging revealed bilateral peripheral signal reduction, with diffuse atrophy (Fig. 3c, d). In the left eye an area with a speckled appearance, of increased and decreased signal, corresponding to the CNV was observed (Fig. 3d). She had serial OCT imaging performed over 9 years which showed initial preservation of macular architecture and only mild intraretinal cystic parafoveal changes in both eyes, with progression to cystoid macular edema in the left and subsequent CNV formation. Loss of outer retinal architecture outside of the foveal center was observed in both eyes (Fig. 3e, f).

#### Electrophysiological assessment

Testing at the age of 23 years revealed an undetectable dim flash rod (DA0.01) ERG and severe reduction of the strong flash ERG (DA10.0) a-waves; the photopic (LA30Hz and LA3.0) ERGs were similarly reduced and showed severe delay bilaterally (Fig. 3). The findings were in keeping with severe generalised rod and cone photoreceptor dysfunction, with bilaterally undetectable pattern ERGs suggesting severe macular involvement.

#### Molecular genetics

The patient was found to harbor the disease-causing *RPGR* variant c.1894_1897delGACA, p.Asp632Lysfs*4, which was segregated to both her mother and her daughter Her mother had a history of nyctalopia. The daughter’s ('*RPGR*-Carrier'). findings are presented below.

### *RPGR*-carrier

The youngest daughter of Patient 5 presented at 5 years old for retinal dystrophy screening, with her parents noting that she was afraid of the dark from early childhood. The patient had a past medical history of bilateral moderate sensorineural hearing impairment and recurrent upper respiratory tract infections.

#### Clinical findings

Her BCVA was 0.20 LogMAR bilaterally. Her anterior segment examination was normal with clear lenses, and slight granular RPE changes at both maculae. A tapetal-like reflex typical of X-linked carrier status was observed (Fig. 3g, h). BCVA was 0.0 LogMAR with spectacle correction. Parents reported visual symptoms that were consistent with nyctalopia and peripheral vision difficulties.

#### Retinal imaging

Extensive changes were observed on FAF (Fig. 3i, j), due to the tapetal-like reflex. Increased reflectivity of the outer retinal bands was observed in an otherwise normal OCT (Fig. 3k, l), in keeping with previous reports [15].

#### Electrophysiological assessment

Ganzfeld ERG recordings (DA0.01, DA10.0, LA30 Hz and LA3.0 ERGs) obtained with lower eyelid skin electrodes revealed no evidence of retinal dysfunction or significant inter-ocular asymmetry and pattern ERGs revealed no evidence of macular dysfunction in either eye.

## Discussion

Herein we presented in detail five cases, with the clinical diagnosis of LCA/EOSRD and the molecular confirmation for disease-causing variants in genes either only very rarely or not previously associated with the disease.

*PRPF8* (OMIM: 607300) encodes for precursor mRNA-processing factor 8, a spliceosome component [16], a gene known to cause autosomal dominant retinitis pigmentosa (ADRP, RP13 MIM: 600059). The *PRPF8* variant (c.5804G > A, p.Arg1935His) identified in our patient, has been previously reported in the heterozygous state in a patient with childhood-onset ADRP [17], as well as in 3 patients with ADRP screened by the Manchester Centre for Genomic Medicine. Recently, in a large EOSRD/LCA genotyping study, a patient with EOSRD was genotyped as *PRPF8* (p.X2336Serext*41, age of onset: 3 years old) [7]. However the presented clinical information is limited to age of onset and the clinical diagnosis of EOSRD/LCA. The patient described in the current report is the youngest patient affected with *PRPF8*-associated disease (including the identified families in Moorfields Eye Hospital, n = 15) [18], and the first detailed clinical report in the literature.

*PRPH2* (OMIM: 179605) is a five-exon gene encoding peripherin-2, a cell surface glycoprotein in the outer segments, with an essential role in disc morphogenesis [19–21]. *PRPH2* is associated with a wide range of clinical phenotypes, including: AD central areolar choroidal dystrophy (CACD, MIM: 613105), AD macular pattern dystrophy (MPD, MIM: 169150), AD vitelliform macular dystrophy (MIM: 608161), AD/AR retinitis punctata albescens (MIM: 136880), AD/AR RP (MIM: 608133) and AR LCA/EOSRD (LCA18, MIM: 608133). Homozygous variants in *PRPH2* have been reported in two individuals with LCA (c.637 T > C, p.C213R and c.554 T > C, p.L185P) [22]. Family members heterozygous for the variant were asymptomatic but showed butterfly-shaped MPD on clinical examination [22]. In a pedigree harboring the variant p.Gln238Ter, intrafamilial variability was also observed; with the heterozygous members exhibiting CACD and MPD, and the patient in the homozygous state exhibiting LCA/EOSRD [23]. The reported homozygous variant herein is novel to the best of our knowledge (c.620_627delinsTA, p.Asp207_Gly208del). In our pedigree, clinical examination and detailed retinal imaging was performed in heterozygous parents, with only a small drusenoid deposit identified in the one eye of the father of patient 2 at age of 28 years. We provide the detailed phenotype of the fourth patient described with LCA18, and consolidate *PRPH2* as a cause of LCA; with previous descriptions often lacking clinical detail.

*RP1* (OMIM 603937) encodes for oxygen-regulated photoreceptor protein 1 (ORP1), and has been associated with ADRP and ARRP (RP1, MIM: 180100) [24, 25]. The majority of cases in the literature follow an AD mode of inheritance [26]. Previous studies reported that the development of ADRP and ARRP depended on the location of the *RP1* variants [25, 26]. Nonsense-mediated mRNA decay (NMD) is an mRNA surveillance mechanism that leads to a degradation of the transcripts with introns in the 3′ untranslated region, preventing the synthesis of truncated proteins that may have toxic effects, such as dominant negative interactions [27, 28]. Chen et al. suggested four classes of truncation variants in the *RP1* gene with different effects on the inheritance mode of RP: (i) Class I are NMD-sensitive truncations located in exons 2 and 3, (ii) Class II are NMD-insensitive truncations located in a region spanning approximately p.500 to p.1053 in exon 4 (deleterious—dominant negative effect), (iii) Class III are NMD-insensitive truncations located in the regions p.264 to p.499 and p.1054 to p.1751 (loss-of-function, ARRP), and iv) Class IV includes NMD-insensitive truncations located after p.1816 in exon 4, with the resulting truncated proteins expected to have normal function [25]. The identified novel variant in the current study (c.4147_4151delGGATT) is a class III variant according to the aforementioned classification, and is expected to cause AR disease in the homozygous state and carriers to be asymptomatic, in agreement with the presented pedigree. Initially class IV variants were perceived as non-pathogenic and only recently have been associated with AR macular dystrophy and cone-rod dystrophy [29]. Kabir et al. reviewed all published *RP1* variants (2016) and observed that the heterozygous variants responsible for ADRP resided between amino acid residues 617–1551, and the homozygous variants responsible for ARRP cluster in two regions: amino acids 193–736 and amino acids 1243–1890 [26]. The overlap of ADRP and ARRP phenotypes for variants in the amino acid residues 617–736 and 1243–1551, lead to the speculation that the nature of the variant and not only its location dictates the inheritance pattern. Recently one case of EOSRD (age of disease onset: 4 years old) was reported with compound heterozygous class III variants [7]. Our truncating variant (within the 1243–1551 amino acids group), presented with the earliest disease onset of all the reported cases in the literature and the clinical diagnosis of LCA/EOSRD, in both siblings; further extending the genetic and clinical spectrum and validating *RP1* as a rare cause of LCA/EOSRD.

*RPGR* (OMIM: 312610) encodes for retinitis pigmentosa GTPase regulator and is a nineteen exon gene that gives rise to two alternatively spliced retinal isoforms, encoded by exons 1–19 and 1–15 (+ part of intron 15) respectively [30]. The latter isoform, also known as exon open reading frame 15 (ORF15), is the most highly expressed retinal variant and a mutational hotspot [31–33]. Most disease-causing variants in *RPGR* result in RP (RP3, MIM: 300029) [34], but those leading to cone and cone-rod dystrophy (MIM: 304020) are preferentially located at the 3′ end of the ORF15 region [35, 36]. *RPGR* variants lead also to atrophic macular degeneration (MIM: 300834) and ciliopathy (MIM: 300455) [36]. Identical intrafamilial sequence variants in *RPGR* may lead to distinctly different phenotype [37, 38]. Our *RPGR* patient is a female, and while female *RPGR* carriers can be affected to a variable extent, depending on X-chromosome inactivation ratios, carriers are usually mildly affected or unaffected [39], whereas some can progress to moderate or severe vision loss after the third decade of life [40]. A high proportion of adult female carriers of XLRP manifest significant ERG abnormalities [41], although typically much milder than in male patients and rarely as severe as in case 5, even in older individuals. In most female heterozygotes, retinal dysfunction is manifest as asymmetrical LA 30 Hz flicker ERG delay and reduction in rod-mediated scotopic strong flash ERG a-waves, the asymmetry being highly unusual for a genetically-determined retinal disorder**.** Phenotypic diversity can be attributed to a certain extend to genetic factors (*i.e.* allelic heterogeneity and genetic modifiers) [38]. Single nucleotide polymorphism (SNP) screen of *RPGR* patients displaying varying disease severity showed that SNPs in *IQCB1* (I393N) and *RPGRIP1L* (R744Q) may be associated with disease severity [38]. Our patient was negative for both those SNPs, however, no study to date has performed whole genome sequencing to identify genome wide modifiers in a cohort of *RPGR* patients. Our patient represents the first report of an *RPGR* variant causing LCA/EOSRD, with the family being another example of marked intrafamilial phenotypic variability.

At present, whilst there are no specific proven treatments for the genotypes discussed herein, several avenues of intervention show promise for molecularly characterized patients, with several challenges and limitations including mode of inheritance and gene size. The AD inheritance of *PRPF8* limits the utility of 'simple' gene augmentation therapy and requires more complex approaches, including gene editing technology, which are not as advanced. In contrast, adeno-associated viruses (AAV) and compacted DNA nanoparticles carrying *PRPH2* have been successfully used to mediate gene transfer and improvement in the *rds*^−/−^ and *rds*^+/−^ mouse models [42]. Successful integration and material transfer of donor- or stem cell-derived cone photoreceptors in Prph2^rd2/rd2^ murine models of the disease are also promising [43]. A pharmacological approach, with inhibition of the overactive poly-ADP-ribose polymerase (PARP), with the PARP inhibitor PJ34, has demonstrated a decrease in the levels of poly-ADP-ribosylation and photoreceptor cell death, in this same model [44]. For recessive *RP1* disease, gene supplementation may be a potential therapeutic intervention; however, *RP1* consists of 6468 base pairs, making it too large for current AAV vectors [45]. Human treatment trials of gene replacement therapy are already underway for *RPGR*-associated RP (NCT03252847, NCT03116113, and NCT03316560).

The current study provides clinical and genetic evidence, which extends the phenotypic spectrum of *PRPF8-*, *PRPH2-, RP1-*, and *RPGR-*associated disease, and the genotypic spectrum of LCA/EOSRD. All cases had disease onset within the first year of life, with lifelong morbidity for those patients. Molecular genetics, segregation results, and retinal and functional phenotypes are presented in detail, the latter revealing unexpectedly severe disease in some cases. The study emphasizes the critical need for molecular confirmation of disease but also highlights phenotypic variability and the need for detailed electrophysiological and retinal characterization. Comprehensive phenotyping of patients with inherited retinal disease is of vital importance to identify suitable candidates and to enable benefit from future therapeutic advancements.

## Supplementary Information

**Additional file 1**. Supplementary Table: Annotation of Leber Congenital Amaurosis/Early-Onset Severe Retinal Dystrophy Causing Variants.

## Data Availability

All data generated or analysed during this study are included in this published article and its supplementary information files.
