# Supplementary material for: Extending the phenotypic spectrum of PRPF8, PRPH2, RP1 and RPGR, and the genotypic spectrum of early-onset severe retinal dystrophy
Source: Orphanet J Rare Dis. 2021 Mar 12;16:128. doi: 10.1186/s13023-021-01759-8 (PMC7953775; doi:10.1186/s13023-021-01759-8)
Supplement: Supplementary file 1 — Additional file 1. Supplementary Table: Annotation of Leber Congenital Amaurosis/Early-Onset Severe Retinal Dystrophy Causing Variants. [file 13023_2021_1759_MOESM1_ESM.docx]

| **Supplementary Table: Annotation of Leber Congenital Amaurosis/Early-Onset Severe Retinal Dystrophy Causing Variants** | | | | | | | | | |
| --- | --- | --- | --- | --- | --- | --- | --- | --- | --- |
| **Pedigree** | **Gene** | **c.DNA** | **Protein** | **Variant ID** | **gnomAD  Allele Count** | **CADD** | **Inheritance Mode** | **Segregation** |  |
|  |  |  |  |  |  |  |  |  |  |
| **GC23684** | ***PRPF8*** | c.5804G>A | p.Arg1935His | chr17-1558827-C-T | NA | 35 | Autosomal  Dominant | Segregated to parents, both negative |  |
|  |  |  |  |  |  |  |  |  |  |
| **GC21703** | ***PRPH2*** | c.620_627delinsTA | p.Asp207_Gly208del | chr6-42672304-GACGCCGT-TA | NA | 18.39 | Autosomal  Recessive | Segregated to parents, both positive carriers, first cousins |  |
|  |  |  |  |  |  |  |  |  |  |
| **GC21938** | ***RP1*** | c.4147_4151delGGATT | p.Gly1383* | chr8-55540587-ATGGAT-A | 1/250,764 | 28.5 | Autosomal  Recessive | Segregated to mother (unaffected carrier) and affected brother - tested positive |  |
|  |  |  |  |  |  |  |  |  |  |
| **GC17432** | ***RPGR*** | c.1894_1897delGACA | p.Asp632Lysfs*4 | chrX-38146354-TTGTC-T | NA | 28.1 | X-linked | Segregated to mother and daughter |  |
|  |  |  |  |  |  |  |  |  |  |

CADD: Combined Annotation Dependent Depletion

Variant annotation of all 4 variants reported in the study. The Combined Annotation Dependent Depletion (CADD) (Rentzsch et al 2019) score is indicative of pathogenicity. The gnomAD exomes dataset contains 250,764 alleles, the gnomAD genomes dataset contains 31,416 alleles. Three variants were not found in gnomAD exomes/genomes despite good coverage, and the other one was reported 1/250,764; in keeping with them being very rare and supporting disease-causation.
